# Lung Perfusion Imaging in Tetralogy of Fallot: A Case Report

**DOI:** 10.4274/mirt.04909

**Published:** 2018-10-09

**Authors:** Artor Niccoli Asabella, Alessandra Cimino, Corinna Altini, Valentina Lavelli, Giuseppe Rubini

**Affiliations:** 1 Bari University Aldo Moro, Unit of Nuclear Medicine, Bari, Italy

**Keywords:** Lung scintigraphy perfusion, congenital heart disease, tetralogy of fallot, right-to-left shunt

## Abstract

Congenital heart diseases, such as tetralogy of fallot (TOF), are the most common human birth defects that may cause pulmonary diseases. Lung perfusion scintigraphy (LPS) has an important role in evaluating pulmonary involvement in patients with these defects, both as part of the diagnostic work-up and for follow-up to guide best therapeutic strategy. Herein, we report a 10-year-old female patient with TOF who underwent LPS two years after cardiac surgery. The scan showed hypo-perfusion of the left respect to the right lung and abnormal uptake of Tc-99m-macroaggregated albumin in the kidneys and spleen, revealing the presence of a right-to-left shunt, and the necessity for further cardiac surgery. This case is a demonstrative example of the usefulness of LPS in patients with TOF, allowing an accurate evaluation of the best therapeutic strategy with the benefits of low radiation exposure, lack of side effects, reproducibility, management ease and good patient compliance.

## Abstract (tr)

Fallot tetralojisi (TOF) gibi konjenital kalp hastalıkları, pulmoner hastalıklara neden olabilecek en yaygın doğumsal kusurlardandır. Akciğer perfüzyon sintigrafisi (LPS), bu hastalarda akciğer yükünün değerlendirilmesinde, en iyi tedavi stratejisini belirlemede ve takip sürecinde önemli bir role sahiptir. Bu yazıda 10 yaşında kardiyak cerrahiden iki yıl sonra LPS uygulanan TOF’lu bir kız hasta bildirilmektedir. Sağ akciğer sol altta hipo-perfüzyon ile birlikte böbreklerde ve dalakta anormal Tc-99m-makro albümin tutulumu, sağdan sola şant varlığını ve ileri kardiyak cerrahinin gerekliliğini ortaya koydu. Bu olgu, LPS’nin TOF hastalarında yararının bir örneği olup, bu teknik düşük radyasyon maruziyeti, yan etkisinin olmaması, tekrarlanabilirliği, kolay yöntemi ve iyi hasta uyumu ile en iyi tedavi stratejisinin doğru olarak değerlendirilmesini sağlar.

## Figures and Tables

**Figure 1:**
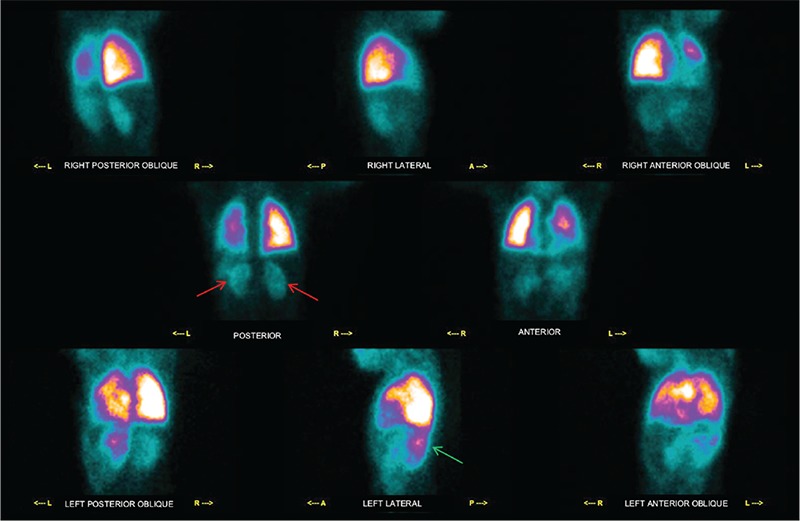
Congenital Heart diseases (CHD), such as tetralogy of fallot (TOF), are the most common human birth anomalies with severity depending on the anatomical defect with subsequent different clinical impact and management (1). CHD may be associated with pulmonary hemodynamic defects so it is important to know lung perfusion status, since its asymmetry is a predictor of exercise capacity and outcome (2). Lung perfusion scintigraphy (LPS) is the gold standard for qualitative and semi-quantitative evaluation of pulmonary perfusion in patients with CHD (3). We report a case of a 10-year-old female patient with cyanotic spells, hypoxia and squatting episodes since birth that progressively increased during the years. A computed tomography angiography performed with a presumptive diagnosis of CHD revealed a cardiac anatomy as in TOF and the patient was admitted to pediatric cardiac surgery clinic according to the European Society of Cardiology guidelines. Her post-operative second year follow-up physical examination revealed clubbing and hypoxia as well as continuous murmur over the chest. Her abdomen was soft with normal bowel sounds and no organ enlargement. The chest X-ray suggested lung inflammatory interstitial disease without focal lesions, pleural effusion or cardiomegaly, her blood work-up revealed Hb: 10.5 gr/dL (normal values 11-13 g/dL), Htc: 36.8% (normal values 35-42%), and fibrinogen protein: 630 mg/dL (normal values 150-400 mg/dL). The patient underwent LPS by intravenous injection of 111 MBq Tc-99m-macroaggregated albumin to evaluate pulmonary hemodynamics and impairment. This figure depicts images of the thorax-abdomen acquired in all the 8 executable planar projections (i.e. right posterior oblique, right lateral, right anterior oblique, posterior, anterior, left posterior oblique, left lateral, left anterior oblique). LPS showed 80% radiopharmaceutical uptake in the lungs and the remnant 20% in the kidneys (red arrows) and spleen (green arrows). This finding supported the presence of a right-to-left shunt, and the patient was kept in close follow-up to be scheduled for further cardiac surgery.

**Figure 2:**
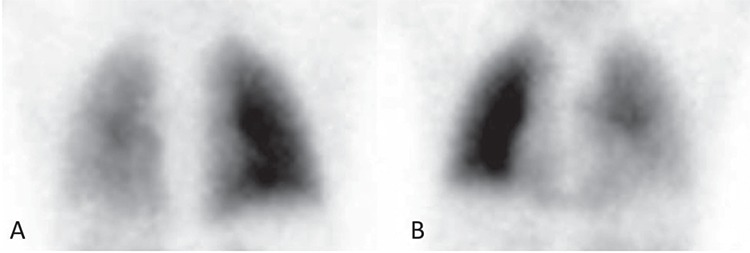
LPS is a functional diagnostic tool that records the distribution of pulmonary arterial blood flow. The most common clinical indications for LPS are to detect pulmonary embolism, to quantify differential pulmonary perfusion before surgery or in chronic disorders, to evaluate the cause of pulmonary hypertension and assessment for lung transplantation. In patients with CHD, LPS evaluates the co-existence of congenital heart and lung hemodynamic defects such as cardiac shunt, pulmonary arterial stenosis, arteriovenous fistula and their treatment (4). LPS can depict normal symmetrical perfusion, unilateral absent or decreased perfusion, or multiple segmental abnormalities in patients with CHD. It allows to define the presence of a right to left shunt due to the presence of aortic-pulmonary collaterals vessels, associated with cyanogenic CHDs such as TOF (5). This LPS figure shows the thorax in detail, in the posterior (A) and anterior (B) projections, which clearly depicts hypo-perfusion of the left lung as compared to the right.

**Figure 3:**
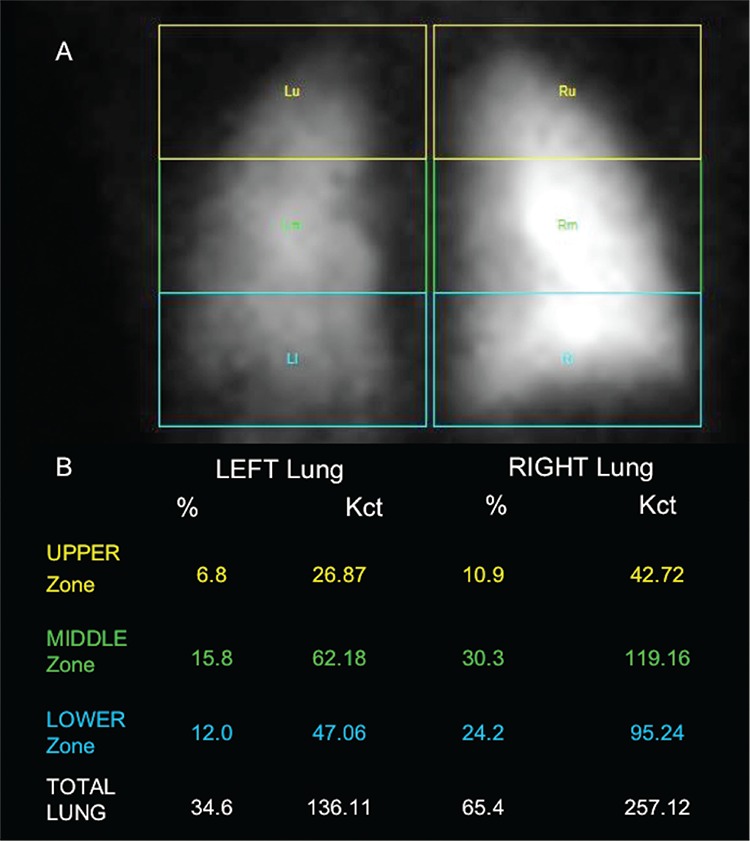
Semi-quantitative analysis identified significant reduction of radiopharmaceutical uptake in the upper, middle and lower regions of the left lung with respect to the right (A) and confirmed left lung hypo-perfusion (B), thus requiring further surgery. LPS has an important role in the diagnosis, as part of work-up for better patient management, and during follow-up to confirm surgery or therapy success in patients with CHD (6). Our case is a demonstrative example of LPS usefulness in the evaluation of lung hemodynamic effects in pediatric patients with TOF, allowing an accurate evaluation of the best therapeutic strategy with benefits of low radiation exposure, lack of adverse effects, reproducibility, management ease and good patient compliance.
